# *Achromobacter* buckle infection diagnosed by a 16S rDNA clone library analysis: a case report

**DOI:** 10.1186/1471-2415-14-142

**Published:** 2014-11-24

**Authors:** Fumika Hotta, Hiroshi Eguchi, Takeshi Naito, Yoshinori Mitamura, Kohei Kusujima, Tomomi Kuwahara

**Affiliations:** Department of Ophthalmology, Institute of Health Biosciences, The University of Tokushima Graduate School, 3-18-15, Kuramoto-cho, Tokushima, 770-8503 Japan; Kusujima Eye Clinic, 255-1, Shimobun, Kinsei-cho, Shikokuchuo-shi, 799-0111 Japan; Department of Microbiology, Faculty of Medicine, Kagawa University, 1750-1, Ikedo, Miki-cho, Kida-gun, 761-0793 Japan

## Abstract

**Background:**

In clinical settings, bacterial infections are usually diagnosed by isolation of colonies after laboratory cultivation followed by species identification with biochemical tests. However, biochemical tests result in misidentification due to similar phenotypes of closely related species. In such cases, 16S rDNA sequence analysis is useful. Herein, we report the first case of an *Achromobacter*-associated buckle infection that was diagnosed by 16S rDNA sequence analysis. This report highlights the significance of *Achromobacter* spp. in device-related ophthalmic infections.

**Case presentation:**

A 56-year-old woman, who had received buckling surgery using a silicone solid tire for retinal detachment eighteen years prior to this study, presented purulent eye discharge and conjunctival hyperemia in her right eye. Buckle infection was suspected and the buckle material was removed. Isolates from cultures of preoperative discharge and from deposits on the operatively removed buckle material were initially identified as *Alcaligenes* and *Corynebacterium* species. However, sequence analysis of a 16S rDNA clone library using the DNA extracted from the deposits on the buckle material demonstrated that all of the 16S rDNA sequences most closely matched those of *Achromobacter* spp. We concluded that the initial misdiagnosis of this case as an *Alcaligenes* buckle infection was due to the unreliability of the biochemical test in discriminating *Achromobacter* and *Alcaligenes* species due to their close taxonomic positions and similar phenotypes. *Corynebacterium* species were found to be contaminants from the ocular surface.

**Conclusions:**

*Achromobacter* spp. should be recognized as causative agents for device-related ophthalmic infections. Molecular species identification by 16S rDNA sequence analysis should be combined with conventional cultivation techniques to investigate the significance of *Achromobacter* spp. in ophthalmic infections.

## Background

A 16S ribosomal DNA (rDNA) clone library analysis was performed for microbiological diagnosis in a clinical case of buckle infection. This type of analysis has previously been applied to a number of environmental samples to examine the microbial diversity within an ecological niche [1–6]. In clinical settings, it can be used to determine the microbial compositions of specimens, which would be beneficial to human health and would further our understanding of the pathological manifestations due to chronic infections [7–9]. In addition, in acute infections, causative bacteria are expected to be readily identified from the predominant sequences in specimens when a 16S rDNA clone library analysis is employed.

Buckle infection is a rare postoperative complication of retinal detachment. It generally occurs in the late stages of postoperative course. Although resident bacteria on the ocular surface, such as *Staphylococcus aureus* and *Staphylococcus epidermidis*, have been reported as the causative pathogens [10–12], environmental bacteria such as *Pseudomonas aeruginosa* or *Stenotrophomonas maltophilia* can also cause infections [12–15]. Some of the previous articles describing device-related ophthalmic infections reported isolation of a single pathogen. Considering that we currently know relatively very little about the diversity of microorganisms in nature [16], culture-independent molecular approaches to detect the causative agents may be useful for diagnosis of buckle infections. More than one pathogenic strains and unreported environmental strains could be detected if the molecular genetic approach were applied to those cases. Herein, we report the first case of an *Achromobacter* species-associated buckle infection diagnosed by use of a 16S rDNA clone library analysis.

## Case presentation

**Figure 1:**
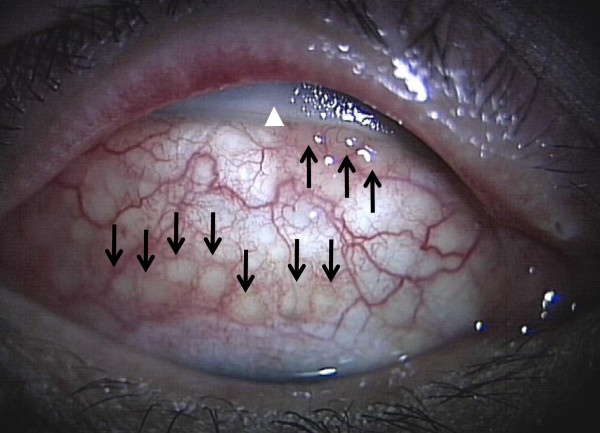
**Pre-operative anterior segments photograph.** The patient is looking downward. Conjunctival fistula in the upper quadrants and large yellowish conjunctival follicles (black arrows) around the exposed buckle material (white arrowhead) can be observed.

A 56-year-old woman complained of purulent discharge and conjunctival hyperemia in her right eye. These symptoms began several months prior to the first visit to our hospital. Eighteen years prior, she had received an uneventful scleral buckling surgery using a solid silicone tire in her right eye for rhegmatogenous retinal detachment. Thirteen years after the surgery, she was administered oral cephem antibiotics once on suspicion of a buckle infection. Although the symptoms temporarily improved, chronic inflammation persisted for several years. Because subsequent topical quinolone and topical steroid treatments were ineffective, she visited our hospital for rigorous diagnosis and radical treatment. On the first visit, the best-corrected visual acuity was 20/200 in the right eye. Observation by a slit lamp microscope revealed conjunctival hyperemia, purulent discharge, and episcleritis. A conjunctival fistula was also observed in the upper quadrants, and large yellowish conjunctival follicles around the exposed buckle material were present (Figure  1). After examination, we removed the buckle material based on the diagnosis of recurrent buckle infection.

Pre-operatively, *Alcaligenes* and *Corynebacterium* species were isolated from the eye discharge. The bacterial identification and drug susceptibility tests were performed automatically using a MicroScan WalkAway 96 SI (Siemens Healthcare Diagnostics, Tokyo, Japan). During the surgery, a 120° solid silicone tire was removed and the scleral bed was irrigated with 0.5% moxifloxacin ophthalmic solution. Post-operatively, 300 mg/day of oral cefdinir was administered for 3 days, and both 0.5% moxifloxacin ophthalmic solution and 0.1% betamethasone sodium phosphate ophthalmic solution were administered 5 times daily for 2 weeks. After removal of the silicone tire, the symptoms improved rapidly. Retinal detachment had not recurred at this point.

**Figure 2:**
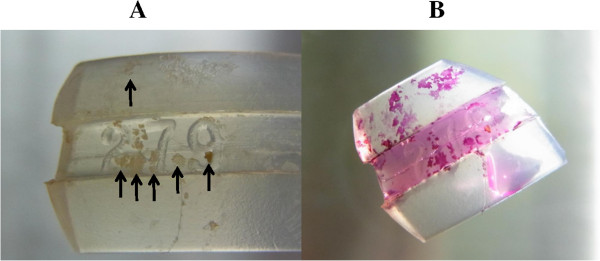
**Pictures of the buckle material. (A)** Buckle material immediately after the extraction. Many yellowish-white deposits (arrows) on the surface of the buckle material can be observed. **(B)** Ruthenium red staining. Deposits were stained red by ruthenium red for scanning electron microscopy.

Many small yellowish-white deposits were found on the surface of the removed buckle material (Figure  2A). Gram staining of the deposits showed a large number of gram-negative rods. *Alcaligenes* and *Corynebacterium* species were also isolated from the buckle material. Species identification and drug susceptibility results were obtained through laboratory procedures identical to those performed preoperatively. The drug susceptibility of the *Alcaligenes* strain isolated from the buckle was identical to that of the strain preoperatively isolated from the eye discharge (Table 1). In the case of *Corynebacterium*, there was a definite discrepancy in the drug susceptibilities between the strains obtained pre- and postoperatively; the strain isolated from the eye discharge was resistant to cephalosporin, but the strain isolated from buckle depositions was susceptible to all antibiotics tested (Table 2). Microbiological examination of the removed buckle material indicated that the causative pathogen is a bacterium that belongs to the family Alcaligenaceae. We employed a 16S rDNA clone library analysis to identify the causative bacterium at the species level and to assess the possibility of the involvement of other uncultured species in the buckle infection. Initially, the buckle material was divided into two pieces, and one piece was stained with ruthenium red for examination by scanning electron microscope (SEM) (Figure  2B). The other piece was placed into 15 mL of phosphate-buffered saline (PBS) and sonicated repeatedly using a VialTweeter (Hielscher Ultrasonics Gmbh, Berlin, Germany) at 60 W for 15 min at room temperature. PBS was replaced twice, and the final sonicate was used for DNA extraction.

**Table 1:** **The drug susceptibilities of the strain of** ***Alcaligenes*** **sp. and the strain of** ***Corynebacterium*** **sp.**

	***Alcaligenes***		***Corynebacterium***	
Antibiotic	Discharge	Buckle	Discharge	Buckle
Ampicillin	S	S	-	-
Penicillin G	R	R	-	-
Cefmenoxime	-	-	R	S
Ceftizoxime	R	R	R	S
Cefroxime	-	-	R	S
Cefepime	-	-	R	S
Cefpodoxime pivoxil	-	-	R	S
Azithromycin	-	-	R	S
Gentamicin	R	R	-	-
Tobramycin	R	R	-	-
Dibekacin	I	I	-	-
Arbekacin	I	I	R	S
Levofloxacin	I	I	S	S
Ciprofloxacin	S	S	S	S
Chloramphenicol	S	S	-	-
Imipenem/cilastatin	S	S	S	S
Meropenem	S	S	S	S

Hyphen: not performed. S: susceptible. I: intermediate. R: resistant.

Although the two strains of *Alcaligenes* sp. show the same profiles, the two strains of *Corynebacterium* sp. show different profiles.

**Table 2:** **Summary of 16S rDNA clone library analysis of the infected buckle material**

Sequence type ^a)^	No. of clone ^b)^	Best match ^c)^
ST1	12	*Achromobacter spanius* strain LMG 5911 (631/633; 99.7%)
ST2	1	*Achromobacter spanius* strain LMG 5911 (628/631; 99.5%)
ST3	1	*Achromobacter spanius* strain LMG 5911 (628/632; 99.4%)
ST4	1	*Achromobacter spanius* strain LMG 5911 (627/630; 99.5%)
ST5	1	*Achromobacter spanius* strain LMG 5911 (630/632; 99.7%)
ST6	1	*Achromobacter spanius* strain LMG 5911 (623/625; 99.7%)
ST7	1	*Achromobacter spanius* strain LMG 5911 (627/628; 99.8%)
ST8	1	*Achromobacter spanius* strain LMG 5911 (630/632; 99.7%)
ST9	1	*Achromobacter spanius* strain LMG 5911 (627/628; 99.8%)
ST10	1	*Achromobacter spanius* strain LMG 5911 (624/625; 99.8%)

^a)^Twenty-one 16S rDNA sequences obtained (631 bp of high quality sequence) are classified basing on SNIPs.

^b)^Number of sequence belonging to each sequence type is shown.

^c)^Top hit microorganisims by which Blastn search of each sequence type indicated are listed.

The number in parenthesis shows identical base (bp)/alignment length (bp) to 16S rDNA from indicated species.

**Figure 3:**
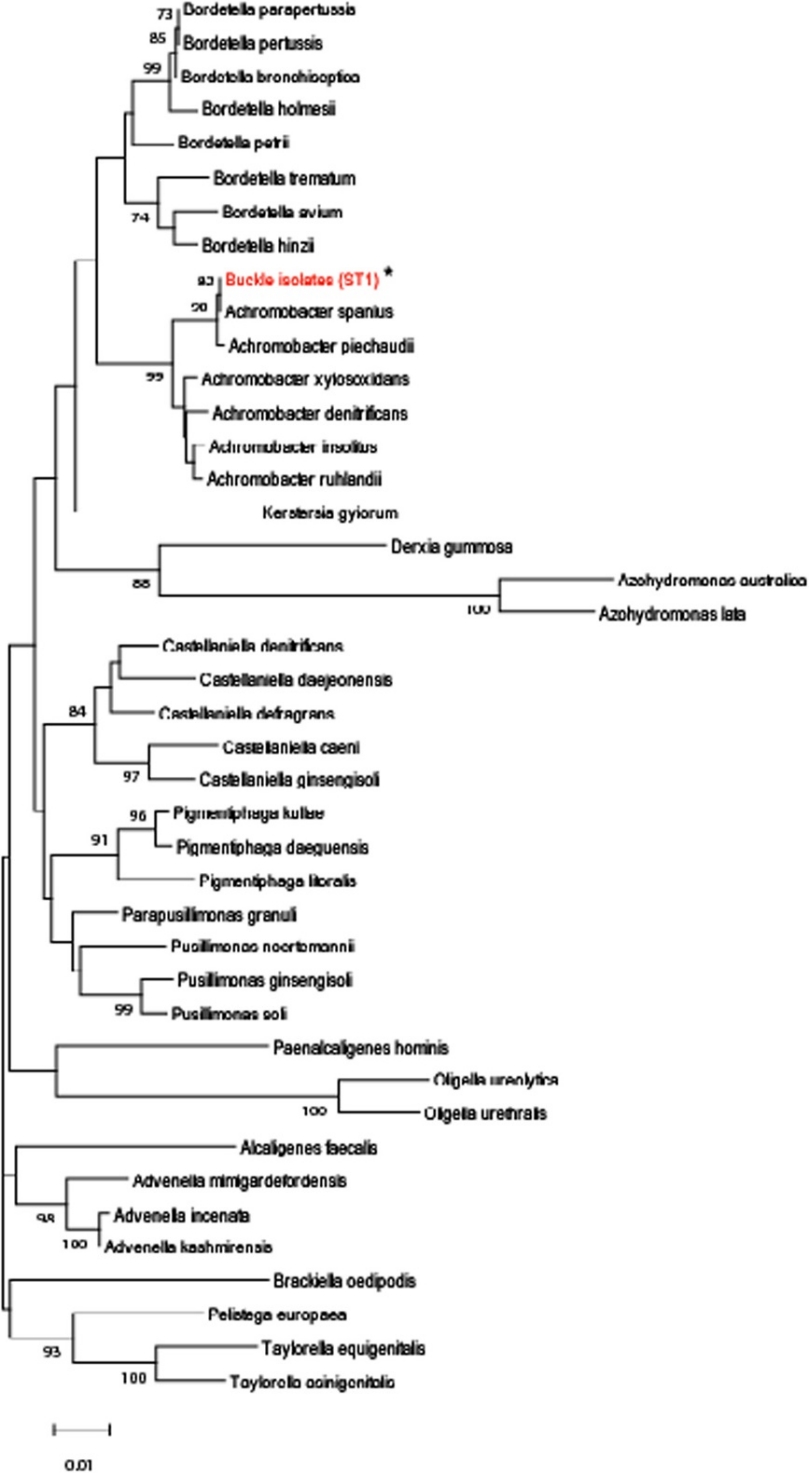
**Phylogenetic relationship between the isolate from buckle material and other members of the family** ***Alcaligenaceae*** **.** Only the most predominant ST1 sequence (indicated by red and asterisks) was analysed. The tree was constructed using the neighbour-joining algorithm. Numbers at nodes are bootstrap percentages based on 1,000 replications; only values >70% are shown. Bar, 0.01 substitutions per nucleotide position.

**Figure 4:**
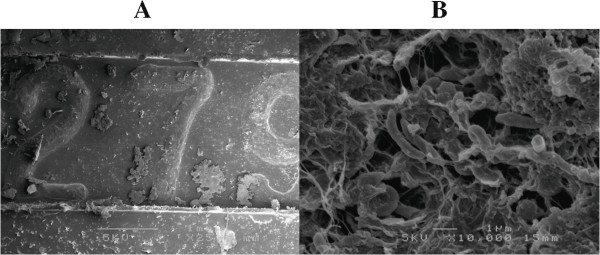
**Scanning electron microscopic images of the buckle material. (A)** Low magnification. Deposits attached after fixation by glutaraldehyde. **(B)** High magnification. Numerous rod-shaped bacteria surrounded by biofilm-like material are observed.

Bacterial DNA was extracted from 200 μL of the final PBS sonicate using Extrap Soil Kit Plus ver.2 (Nippon Steel Kankyo Engineering Co., Ltd., Tokyo, Japan). The 16S rDNA gene fragments were amplified with the purified DNA as a template and a universal eubacterial 16S rDNA primer set, 27f (5'-AGAGTTTGATCMTGGCTCAG-3') and Bac1392R (5'-ACGGGCGGTGTGAC-3'). After cloning the amplified products, the sequences were obtained from 24 clones using 27f as the sequencing primer. The low-quality sequences (Phred score <15) were trimmed, and the sequences were analysed for homology to NCBI database sequences using the Blast program. Of the 24 clones, high-quality sequences were obtained from 23 clones, but two of these were from the genomic regions other than 16S rDNAs. All of the partial 16S rDNA sequences obtained from 21 clones showed the best match to those of *Achromobacter* species (Table  2; identity ranged from 99.4—99.8% over 99% of alignments with query sequences). It is likely that the isolates initially identified as *Alcaligenes* spp. were in fact *Achromobacter* spp. This misidentification was probably due to the low discriminatory power of the biochemical test for the species in the family Alcaligenaceae. Single nucleotide polymorphisms (SNPs) were observed among the sequences (12 sequences were identical). These SNPs might indicate that several different *Achromobacter* strains were present in biofilms on the buckle material, although this was only the sequence diversity among the ribosomal RNA operons in a single *Achromobacter* chromosome. To further refine the identification of causative bacterial species, the most predominant 16S rDNA sequences obtained were aligned with those from 43 reference species (obtained from Ribosomal Database Project ver. 10) in the family Alcaligenaceae. We aligned the 613-bp regions encompassed within well-conserved regions using the Clustal W program to adjust the positions to be compared. All 21 of the sequences were phylogenetically positioned closely with the sequences from *Achromobacter spanius* (Figure  3)*.* Based on these results, we conclude that an *Achromobacter* sp. closely related to *A. spanius* was the causative agent in this case.SEM of the buckle material showed numerous rod-shaped bacteria surrounded by a biofilm-like material, consistent with our conclusions from the 16S rDNA clone library analysis (Figure  4, A and B).

## Conclusions

In clinical settings, cultivation and phenotypic tests of isolated bacteria employing traditional culture techniques is the first step in diagnosis of infectious diseases. In this case, we aimed to identify the causative pathogens for buckle infection by culturing the eye discharge and buckle material. These cultures resulted in the successful isolation of the two candidates, *Alcaligenes* and *Corynebacterium* species. We surmised that *Corynebacterium* spp. were a contaminant as they are one of the resident bacteria on the ocular surface [17], and *Corynebacterium* isolates from the discharge and buckle material showed different antimicrobial susceptibilities. Therefore, these different strains of *Corynebacterium* were most likely from the ocular surface. Correspondingly, the 16S rDNA sequences derived from *Corynebacterium* spp. were not identified in 16S rDNA clone library analysis. We presume that the *Corynebacterium* spp. were washed away by irrigation during surgery and sonication because they only attached to the surface of the buckle material and not embedded within biofilm.

Although *Alcaligenes* spp. were initially considered to be a causative agents, we had doubts about the microbiological identification based on the following observations. First, the isolate in this case showed resistance to aminoglycosides while the majority of *Alcaligenes* species have been reported to be susceptible to gentamicin [18]. Second, the taxonomy of the family Alcaligenaceae is continually revised and updated and the biochemical test is unreliable in discriminating *Alcaligenes* and *Achromobacter* due to their close phylogenetic relationship [19]. Device-related biofilm infections are often caused by opportunistic environmental pathogens and are often polymicrobial. The frequent discrepancy between direct microscopic counts and the number of culturable bacteria from environmental samples is one of several indications that we currently know very little about the diversity of microorganisms in nature [16]. In addition, precise species identification is typically problematic in environmental isolates. Therefore, we employed a 16S rDNA clone library analysis to precisely classify the isolate at the species level and to test the possibility that the biofilm in this case was polymicrobial and contained uncultivable environmental bacteria. Although 16S rDNA clone library analysis using 24 clones is insufficient for excluding the presence of other pathogenic strains, our results show that this case was buckle infection caused by an *Achromobacter* species alone that is closely related to *A. spanius*. To our knowledge, this is the first case report of buckle infection by *Achromobacter* sp. Reliable epidemiological data on bacterial isolates are important for empirical antimicrobial therapy; therefore, precise identification of bacterial species is essential.

Advances in surgery are expected to increase the opportunities for embedding medical devices within the body with a concomitant increase in the risk for device-related infections by opportunistic environmental pathogens. In fact, there are some reports describing *Achromobacter*-related infections from artificial devices such as prosthetic knee joints and contact lenses [20, 21]. Clinicians should take into account the inherent limitations of traditional microbiological assays and combine various approaches to obtain precise diagnoses when necessary. These efforts will likely increase the reliability of epidemiological data in the field of infectious diseases.

The taxonomy of the genus *Alcaligenes* is closely intertwined with that of the genus *Achromobacter* and is frequently revised [19]. *Alcaligenes* has also been isolated from clinical specimens, including ophthalmic samples [12, 22–29]. Coenye *et al.* reported that several isolates identified phenotypically as *Alcaligenes* species belonged to the genus *Achromobacter* based on genetic analysis, and they proposed two novel *Achromobacter* species from these isolates [30]. It is important clinically to discriminate *Alcaligenes* and *Achromobacter* because epidemiological data demonstrate that 72.7% of clinical *Achromobacter* isolates showed multi-drug resistance while all of the *Alcaligenes* isolates tested were susceptible to imipenem, gentamicin, and ciprofloxacin [18]. With regard to the current clinical case, drugs to which *Achromobacter* spp. are potentially susceptible were initially administered, followed by the administration of drugs to which *Achromobacter* spp. are known to be susceptible. However, inflammation around the buckle material continued for several years. SEM observations were indicative of the long clinical course, recurrent symptoms, and *Achromobacter*’s resistance to antibiotic treatment. Therefore, the *Achromobacter*-associated buckle infection case reported here is valuable for considering the epidemiology and antimicrobial therapy of ophthalmic infections. The emergence of device-related infections caused by *Achromobacter* may be intractable, even when efficacious antibiotics are administered.

In conclusion, *Achromobacter* spp. should be recognized as causative agents for device-related ophthalmic infections. Molecular species identification by 16S rDNA sequence analysis should be combined with conventional cultivation techniques to investigate the significance of *Achromobacter* spp. in ophthalmic infections.

### Consent

Written informed consent was obtained from the patient for publication of this case and the accompanying images.
